# Comprehensive evaluation of Luzhou‐flavor liquor quality based on fuzzy mathematics and principal component analysis

**DOI:** 10.1002/fsn3.2796

**Published:** 2022-03-04

**Authors:** Yanbo Liu, Zhangning Qiao, Zhijun Zhao, Xian Wang, Xiyu Sun, Suna Han, Chunmei Pan

**Affiliations:** ^1^ 146228 College of Food and Biological Engineering (Liquor College) Henan University of Animal Husbandry and Economy Zhengzhou China; ^2^ Postdoctoral Programme Henan Yangshao Distillery Co., Ltd. Mianchi China; ^3^ 146228 Henan Liquor Style Engineering Technology Research Center Henan University of Animal Husbandry and Economy Zhengzhou China; ^4^ 146228 Zhengzhou Key Laboratory of Liquor Brewing Microbial Technology Henan University of Animal Husbandry and Economy Zhengzhou China; ^5^ SheDianLaoJiu Co. Ltd. Sheqi China; ^6^ ZhangGongLaoJiu Wine Co. Ltd. Ningling China

**Keywords:** comprehensive evaluation, fuzzy mathematics, Luzhou‐flavor liquor, principal component analysis, quality

## Abstract

Currently, the primary method of identifying high‐ and low‐quality liquors is sensory tasting, which is prone to uncertainty caused by the biases of tasters. To address this problem, this study used color, aroma, taste, and style as four factors affecting the sensory quality of Luzhou‐flavor liquor; determined the weights of each factor; and quantitatively evaluated the sensory quality of five different Luzhou flavor liquor using fuzzy mathematical methods. The volatile aromatic substances in the liquor samples were detected by GC‐MS, and analyzed using principal component analysis. The results obtained from fuzzy mathematics and principal component analysis indicated that the comprehensive evaluation system was scientifically sound and reasonably constructed.

## Graphical abstract

So far, there have been no reports on the application of fuzzy mathematics evaluation method in the sensory evaluation of Luzhou‐flavor liquor and the application of PCA in the comprehensive analysis and evaluation of physical and chemical indexes of liquor. In this experiment, the sensory quality of different liquors was quantitatively evaluated by fuzzy mathematics, GC‐MS was used to detect volatile aroma compounds and principal component analysis of liquor samples, so as to establish a more scientific and objective comprehensive evaluation model of liquor. Fuzzy mathematics was applied in sensory evaluation. Principal component analysis was then employed in the comprehensive analysis of physicochemical indicators. Thereby, a scientific and comprehensive liquor evaluation model was established.

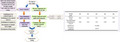

## 1 INTRODUCTION

Chinese liquors are the traditional fermented distilled liquors of China (Zheng & Han, 2016). Luzhou‐flavor liquors, one of the twelve major aromatic liquors in China, have unique flavors and aromas, and account for more than 70% of annual liquor yields in China (He et al., 2019). Luzhou‐flavor liquors are produced from grains with medium‐high temperature, with Daqu being the aroma‐producing agent, and involve continuous distillation of grain ingredients, mixed steaming and mixed burning, solid‐state fermentation, distillation, aging, and blending. Their aromatic compounds are dominated by ethyl caproate, and are produced without the addition of edible alcohols, nonfermented aromatic compounds, or taste‐producing substances (He et al., 2020; Shi et al., 2011).

Fuzzy mathematics is a mathematical theory and method for studying and processing fuzzy phenomena (Wang et al., 2020). It can quantify unclear or unquantifiable boundaries, and scientifically and comprehensively evaluate the multiple quality indicators of the target (Dong & Bi, 2020; Minaev et al., 2020). Sensory evaluation is currently the primary method for determining liquor quality, blending, and flavoring. Traditional sensory evaluation methods generally adopt weighted averaging and total scoring, which are affected by factors like the region, ethnicity, habits, liquor tasting environment, hobbies, and psychological factors of individuals (Cheng et al., 2013; Mukhopadhyay et al., 2013), creating a certain fuzziness. Hence, fuzzy mathematics is suitable for the mathematical and quantitative description/processing of the results of sensory evaluation (Sun et al., 2021). It can eliminate the subjectivity and unilateralism of sensory evaluation (Morales & Boekel, 1997), and thus provide more accurate, objective, reasonable, and scientific evaluation results (Xu et al., 2019; Zou et al., 2019). To date, fuzzy mathematics and sensory evaluation have been combined in research on pit mud (Chang et al., 2018), jams (Shinde & Kulkarni, 2016), yellow rice liquors (Feng et al., 2018), tea (Debjani et al., 2013), sauce (Zhou & Wei, 2019), sausage (Lee & Kwon, 2007), agricultural products and insecticides (Cheng et al., 2021), Radix pseudostellariae healthcare liquors (Zhao et al., 2022), and wine (Song et al., 2021).

Principal component analysis (PCA) is a statistical method that converts multiple variables into a few principal components through dimensionality reduction. This method can, therefore, solve problems involving multiple inter‐related variables (Abdi & Williams, 2010). PCA combines several detection techniques, and is used for the analysis of foods and drugs (Pravdova et al., 2002; Wang et al., 2020). Trace components in liquors are mainly analyzed using gas chromatography–mass spectrometry (GC‐MS; Fan et al., 2019). Research on trace components can reveal the fermentation mechanism of liquors, and is, therefore, vital for understanding their aroma characteristics and overall quality (He et al., 2021; Yan et al., 2020). The results of chromatography are processed by PCA to develop a more objective and effective method for liquor quality evaluation. PCA has been widely applied to the study of beef (Meng et al., 2020), winter jujube (Kou et al., 2021), olive oil (Zhang et al., 2011), mulberry leaf tea (He et al., 2021), and sauce (Feng et al., 2013).

However, till date there is no report of fuzzy mathematics being used for the evaluation of Luzhou‐flavor liquors, nor are there reports of using PCA for the comprehensive analysis of the physiochemical indicators of liquors. Therefore, this study aimed to determine the weights of liquor sensory indicators (color, aroma, taste, and style) and use fuzzy mathematics to quantitatively evaluate the sensory quality of five different Luzhou‐flavor liquor samples. Subsequently, PCA was used for dimensionality reduction of trace components in liquors, statistical analysis, and for the establishment of a comprehensive and scientific mathematical model. The findings of this study will help in the construction of a comprehensive evaluation system for Luzhou‐flavor liquors.

## 2 MATERIALS AND METHODS

### 2.1 Materials and reagents

Luzhou‐flavor liquors with five quality levels (newly produced liquor blended in different proportions with old liquor by the liquor company; the higher the percentage of newly produced liquor, the lower is its quality) were bought from a liquor factory in Henan.

### 2.2 Instruments and machine

Gas chromatograph–mass spectrometer: Shimadzu Corporation, Japan; solid‐phase microextraction device: Merck, USA.

### 2.3 Sensory evaluation

Four evaluation grades of Luzhou‐flavor liquors (Table 1) were set, based on the provisions of sensory properties in GB/T10345‐2007 (A National Standard of the People's Republic of China ‐ Analytical Methods for Liquors). To ensure the accuracy of evaluation and the environmental comfort, we asked the liquor tasters to refrain from drinking, smoking, and eating spicy or irritating foods 24 h before evaluation. They waited for 10 min between evaluations and gargled with clean water during these intervals. Ten tasters (five males and five females) with certificates of liquor evaluation and majoring in liquor‐making engineering, were invited to constitute the evaluation team. Liquor tasters have a background in the systematic theoretical study and practice of brewing and tasting. They are, therefore, trained to earnestly comprehend and understand the relevant evaluation indices, and be objective and fair in their assessment. Based on the grade standards, a single‐factor evaluation involving four indicators (color, aroma, taste, and style) was conducted, and an evaluation table was filled in. With a maximum score of 100, scores of >95, 90–95, 85–90, and 80–85, were considered excellent grade, grade 1, grade 2, and grade 3, respectively.

**TABLE 1:** Sensory rating standard for Luzhou‐flavor liquor

Item	Excellent grade (>95)	First grade (90–95)	Second grade (85–90)	Third grade (80–85)
Color	Colorless or light yellow, clear and transparent, no suspension, no precipitate		Colorless and transparent, slightly turbid, no precipitation	Colorless and transparent, with turbid precipitation
Aroma	Strong compound aroma dominated by ethyl caproate	Strong compound aroma dominated by ethyl caproate	Slight compound aroma dominated by ethyl caproate	No evident compound aroma dominated by ethyl caproate
Taste	Mellow body, moderately sweet and clear, long finish	Mellow and harmonious body, moderately sweet and clear, long finish	Off‐flavor in the body, unclear, uncomfortable finish	Strong off‐flavor, short finish
Style	Typical style in this type of liquors	Slightly typical style in this type of liquors	Not typical style in this type of liquors	No typical style in this type of liquors

### 2.4 Fuzzy mathematical modelling (Lee & Kwon, 2007; Zhou & Wei, 2019)

#### 2.4.1 Establishment of an evaluated target set

Let the evaluated target set of Luzhou‐flavor liquors be B = {M1, M2, M3, M4, M5}, where M1 to M5 represent the samples marked 1–5, respectively.

#### 2.4.2 Establishment of an evaluated factor set

The evaluated factors of the Luzhou‐flavor liquors are U1: color, U2: aroma, U3: taste, and U4: style. The evaluated factor set is, therefore, U = {U1, U2, U3, U4} = {color, aroma, taste, style}.

#### 2.4.3 Establishment of comment set

The evaluation standards for Luzhou‐flavor liquors are based on the four grades (V1–V4) shown in Table 1, which constitute the evaluation set V = {V1, V2, V3, V4} = {excellent grade, grade 1, grade 2, grade 3}.

#### 2.4.4 Determination of evaluation factor weights

Color, taste, aroma, and style were assigned scores of 5, 50, 30, and 15, respectively, so the weight set was X = {0.05, 0.5, 0.3, 0.15}.

#### 2.4.5 Determination of fuzzy matrix

The 10 evaluators scored the liquor samples according to the comment set V. The times of comments given to each index were then plotted in a table. The data in the table were divided by 10 to determine the membership grade R of each of the four factors, for the five liquor samples. A membership grade matrix was obtained by arranging the factors in rows. According to the principle of fuzzy transformation, Y was used as a synthetic evaluation set that contains the products to be evaluated. Therefore, a fuzzy relationship evaluation set was obtained: Y = XR, where X is a weight set and R is a fuzzy matrix. Finally, a comprehensive score matrix T is introduced to process the fuzzy relationship evaluation set Y. According to the specialties of sensory evaluation, let the evaluation grade set be K = {k_1_, k_2_, k_3_, k_4_}. The total score in the fuzzy comprehensive evaluation of liquor samples was T = Y × K, where the evaluation grade set was K = {90, 70, 50, 30}.

### 2.5 GC‐MS analysis conditions

#### 2.5.1 Chromatographic conditions

The chromatographic column used was SHIMADZU Rxi‐5MS capillary column (30 × 0.25 mm, 0.25 μm) with an inlet temperature of 250 ℃ and a column flow of 1.0 ml/min. The sampling method involved splitless injection and heating according to the following program: the starting temperature was 40°C (held for 2 min), was increased at 3.5°C/min to 95°C (held for 2 min), and then increased at 5°C/min to 230°C (held for 10 min); an injection volume of 1 μl was used.

#### 2.5.2 Mass spectrometry conditions

Ion source was from EI and the scan mode used was SCAN mode, the ion source temperature was 220°C, interface temperature was 250°C, electronic capacity was 70 eV, detector voltage was 0.7 kV, solvent delay was by 3.0 min, and scanning range was 30–550 amu.

### 2.6 Data processing

The data obtained were used for statistical analysis and PCA using all‐cause models in Microsoft Office Excel 2016 and SPSS 26.0. The significance level was set at *p* < .05.

## 3 RESULTS

### 3.1 Analysis of the sensory indices of liquor samples using fuzzy mathematics

#### 3.1.1 Results of sensory evaluation of different liquor samples

An evaluation team of ten tasters conducted sensory tasting of the five different liquor samples under special conditions, and evaluated the four indicators of color, aroma, taste, and style, in accordance with the grading criteria developed for dark wine evaluation and single‐factor evaluation (Table 1). The five wine samples were poured into numbered wine glasses by a special person, and evaluated and scored by the ten tasters. The evaluation results were collected, summarized, and statistically analyzed, to produce a statistical table of comprehensive tasting results (counting the number of sensory tasters) (Table 2).

**TABLE 2:** Statistical table of comprehensive evaluation results of 5 liquor samples (statistical number of sensory evaluation)

Sample name	Color	Aroma	Taste	Style
	Excellent First Second Third	Excellent First Second Third	Excellent First Second Third	Excellent First Second Third
M1	9 1 0 0	2 7 1 0	1 4 5 0	2 4 3 1
M2	8 2 0 0	0 5 3 2	0 2 5 3	0 2 6 2
M3	9 1 0 0	6 4 0 0	2 8 0 0	3 6 1 0
M4	9 1 0 0	1 5 4 0	0 3 5 2	1 2 6 1
M5	8 1 0 1	0 4 2 4	0 0 7 3	1 2 3 4

As an example, with respect to the color of the sample M5, 8, 1, 0, and 1 tasters graded it as excellent, 1, 2, and 3, respectively (Table 2). Hence, we obtained U1 = {0.8, 0.1, 0, 0.1}. Similarly, we obtained U2 = {0, 0.4, 0.2, 0.4}, U3 = {0, 0, 0.7, 0.3}, and U4 = {0.1, 0.2, 0.3, 0.4}. Then, a membership grade matrix of the four single factors for the five liquor samples was obtained as follows:

$$R1=\left|\begin{array}{cccc} 0.9 & 0.1 & 0 & 0\\ 0.2 & 0.7 & 0.1 & 0\\ 0.1 & 0.4 & 0.5 & 0\\ 0.2 & 0.4 & 0.3 & 0.1 \end{array}\right|$$

$$R2=\left|\begin{array}{cccc} 0.8 & 0.2 & 0 & 0\\ 0 & 0.5 & 0.3 & 0.2\\ 0 & 0.2 & 0.5 & 0.3\\ 0 & 0.2 & 0.6 & 0.2 \end{array}\right|R3=\left|\begin{array}{cccc} 0.9 & 0.1 & 0 & 0\\ 0.6 & 0.4 & 0 & 0\\ 0.2 & 0.8 & 0 & 0\\ 0.3 & 0.6 & 0.1 & 0 \end{array}\right|R4=\left|\begin{array}{cccc} 0.9 & 0.1 & 0 & 0\\ 0.1 & 0.5 & 0.4 & 0\\ 0 & 0.3 & 0.5 & 0.2\\ 0.1 & 0.2 & 0.6 & 0.1 \end{array}\right|R5=\left|\begin{array}{cccc} 0.8 & 0.1 & 0 & 0.1\\ 0 & 0.4 & 0.2 & 0.4\\ 0 & 0 & 0.7 & 0.3\\ 0.1 & 0.2 & 0.3 & 0.4 \end{array}\right|$$

According to the fuzzy changing principle, the weighted averages were adopted to determine the comprehensive membership grade of the evaluation factors: Y = XR. The evaluation results are as follows:

$$Y1=XR1=\left|\begin{array}{cccc} 0.05 & 0.5 & 0.3 & 0.15 \end{array}\right|\times \left|\begin{array}{cccc} 0.9 & 0.1 & 0 & 0\\ 0.2 & 0.7 & 0.1 & 0\\ 0.1 & 0.4 & 0.5 & 0\\ 0.2 & 0.4 & 0.3 & 0.1 \end{array}\right|=\left\{\begin{array}{cccc} 0\text{.205} & 0\text{.535} & 0\text{.245} & 0\text{.015} \end{array}\right\}$$

$$\text{Similarly},\;Y_{2}=\left\{0.04\;0.35\;0.39\;\;0.22\right\}$$

$$Y_{3}=\left\{\begin{array}{cccc} 0.45 & 0.535 & 0.015 & 0 \end{array}\right\}Y_{4}=\left\{\begin{array}{cccc} 0\text{.11} & 0\text{.375} & 0\text{.44} & 0\text{.075} \end{array}\right\}$$

$$Y_{5}=\left\{\begin{array}{cccc} 0\text{.055} & 0\text{.235} & 0\text{.355} & 0\text{.355} \end{array}\right\}$$

The total score of the fuzzy comprehensive evaluation is T = Y × K. Given that Y_1_ = $\left\{\begin{array}{cccc} 0\text{.205} & 0\text{.535} & 0\text{.245} & 0\text{.015} \end{array}\right\}$ and evaluation grade set K = {90, 70, 50, 30} for M1, we have:

Comprehensive score T_1_ = Y_1_ × K = $\left|\begin{array}{cccc} 0\text{.205} & 0\text{.535} & 0\text{.245} & 0\text{.015} \end{array}\right|$ × $\left|\begin{array}{cccc} 90 & 70 & 50 & 30 \end{array}\right|$ = 68.6

Similarly, T_2_ = 54.2, T_3_ = 78.7, T_4_ = 60.4, and T_5_ = 49.8.

#### 3.1.2 Sensory evaluation results with fuzzy mathematics

During the fuzzy mathematics sensory evaluation, the fuzzy mathematics rationale is used to simulate human thinking and to consider the overall effects of all factors for determining the final result. It eliminates the subjective factors of human evaluators, and thus makes the evaluation process more accurate, objective, and scientific (Pan et al., 2014). Computations showed that the sensory score was the highest in M3, and was significantly higher than other samples. The sensory scores of the five samples were ranked from high to low as M3, M1, M4, M2, and M5. Hence, the sensory evaluation of the five samples can be summarized as M3 > M1 > M4 > M2 > M5. Shinde and Kulkarni (2016) evaluated four different jams available in the market based on a fuzzy‐logic mathematical model using color, flavor, texture, and overall appearance of the jam, as evaluation indicators. The fuzzy mathematical approach was also used to evaluate the quality of a product produced using a new process. Song et al. (2021) evaluated the quality of grape distilled wine using four indicators, namely appearance, color, aroma, and taste.

### 3.2 Construction of quality model for liquor samples

#### 3.2.1 Trace component analysis of liquor samples

The aromatic components of the five liquor samples were detected using GC‐MS. Then, GC‐MS total ion current maps of the representative components in the liquor samples were plotted. The chromatogram components of the Luzhou‐flavor liquors are listed in Table 3. The chromatogram results of the Luzhou‐flavor liquors were not quantitatively analyzed, and were all relative concentrations (%).

**TABLE 3:** Parts of chromatographic components of the Luzhou‐flavor liquor samples

Sample name	Hexanoic acid ethyl ester	Octanoic acid ethyl ester	2‐hydroxy‐Propanoic acid ethyl ester	Butanoic acid hexyl ester	Hexanoic acid butyl ester	2‐methyl Propanol	Butanol	3‐methyl‐Butanol	Hexanol	Furfural	Heptanoic acid	Octanol
M1	33.25	4.15	2.07	5.66	1.01	1.47	7.39	8.57	7.89	0.07	0.64	1
M2	22.99	3.09	2.23	4.58	0.7	1.61	5.92	9.04	6.89	0.2	0.54	0.99
M3	14.45	4.92	0.19	8.04	1.04	0.73	5.55	4.62	6.18	0.56	1.25	2.59
M4	29.95	4.35	1.59	4.77	1.24	1.07	5.73	4.87	6.83	0.1	0.54	0.97
M5	19.1	3.24	4.91	4.97	0.46	0.26	3.41	2.82	5.06	0.32	0.69	1.63

#### 3.2.2 PCA mathematical model

The PCA mathematical model is as follows:

$$F_{1}=a_{11}ZV_{1}+a_{21}ZV_{2}\ldots \ldots +a_{n1}ZV_{m}$$

$$F_{2}=a_{12}ZV_{1}+a_{22}ZV_{2}+\ldots \ldots +a_{n2}ZV_{m}$$

where *a*_1_ *_i_*, *a*_2_ *_i_*, *a_ni_* (*i* = 1, *n*) are the eigenvectors of the eigenvalues in the covariance matrix ∑ from V, and ZV_1_, ZV_2_, ……, ZV_m_ are the standardized values of the original variables. Because the dimensions of the indices are usually different in practical applications, the impact of the dimensions must be eliminated before computation, and the original data must be standardized.

The chromatogram components from the liquors are marked as V_1_, V_2_, V_3_, V_12_. The communality of variables refers to the degree to which a common factor in the original information of each variable can be extracted. The communality of variables extracted by PCA in this study, as shown in Table 4, is above 90% for all variables. This suggests that the loss of information is small, indicating that several common factors extracted in this study can strongly explain these variables.

**TABLE 4:** Common degrees of variables extracted by principal component analysis

	Initial	Extract
*V*_1_	1.000	0.953
*V*_2_	1.000	0.996
*V*_3_	1.000	0.910
*V*_4_	1.000	0.956
*V*_5_	1.000	0.963
*V*_6_	1.000	0.977
*V*_7_	1.000	0.974
*V*_8_	1.000	0.999
*V*_9_	1.000	0.983
*V*_10_	1.000	0.994
*V*_11_	1.000	0.984
*V*_12_	1.000	0.994

The eigenvalues and variance contribution rates of the principal components obtained from the Luzhou‐flavor liquors by PCA are listed in Table 5. Principal components with eigenroots larger than 1 and accumulative contribution rates larger than 80% were selected as the study targets. As shown in Table 5, the eigenroots of the first, second, and third principal components are 6.184, 4.337, and 1.163, respectively (all larger than 1), and their accumulative contribution rates are 51.536%, 87.680%, and 97.371%, respectively, which can efficiently reflect the original data in the indices of the Luzhou‐flavor liquors.

**TABLE 5:** Eigenvalues and variance contributions of the principal components in Luzhou‐flavor liquors

Element	Initial eigenvalue			Extracted sum of squares of load			Sum of squares of rotational loads		
	Total	Percentage of variance	Accumulative contribution rates %	Total	Percentage of variance	Accumulative contribution rates %	Total	Percentage of variance	Accumulative contribution rates %
1	6.184	51.536	51.536	6.184	51.536	51.536	4.664	38.864	38.864
2	4.337	36.144	87.680	4.337	36.144	87.680	3.864	32.198	71.061
3	1.163	9.691	97.371	1.163	9.691	97.371	3.157	26.310	97.371
4	0.315	2.629	100.000						
5	1.058E‐15	8.816E‐15	100.000						
6	4.175E‐16	3.479E‐15	100.000						
7	2.552E‐16	2.126E‐15	100.000						
8	1.279E‐17	1.066E‐16	100.000						
9	−5.521E‐17	−4.601E‐16	100.000						
10	−1.009E‐16	−8.406E‐16	100.000						
11	−6.275E‐16	−5.229E‐15	100.000						
12	−9.963E‐16	−8.302E‐15	100.000						

The eigenvectors were calculated based on the eigenroots of the first three principal components and the load matrix (Table 6). The standardized V_1_, V_2_, V_3_ V_12_ values are marked as ZV_1_ to ZV_12_, respectively. Thus, the principal components are expressed as:

(1) $$F_{1}=‐0.{\text{341ZV}}_{1}+0.150{\text{ZV}}_{2}+0.{\text{167ZV}}_{3}+0.{\text{345ZV}}_{4}‐0.0{\text{43ZV}}_{5}‐0.{\text{128ZV}}_{6}‐0.0{\text{68ZV}}_{7}‐0.0{\text{83ZV}}_{8}‐0.{\text{142ZV}}_{9}+0.{\text{379ZV}}_{10}+0.{\text{372ZV}}_{11}+0.370{\text{ZV}}_{12}$$

(2) $$F_{2}=0.{\text{179ZV}}_{1}‐0.0{\text{16ZV}}_{2}‐0.{\text{224ZV}}_{3}‐0.00{\text{2ZV}}_{4}+0.110{\text{ZV}}_{5}+0.{\text{448ZV}}_{6}+0.40{\text{3ZV}}_{7}+0.{\text{467ZV}}_{8}+0.{\text{395ZV}}_{9}‐0.{\text{157ZV}}_{10}‐0.0{\text{75ZV}}_{11}‐0.{\text{171ZV}}_{12}$$

(3) $$F_{3}=0.{\text{287ZV}}_{1}+0.{\text{857ZV}}_{2}‐0.{\text{668ZV}}_{3}+0.{\text{434ZV}}_{4}+0.{\text{879ZV}}_{5}+0.0{\text{79ZV}}_{6}+0.{\text{455ZV}}_{7}‐0.0{\text{88ZV}}_{8}+0.{\text{396ZV}}_{9}‐0.0{\text{36ZV}}_{10}+0.{\text{299ZV}}_{11}+0.{\text{143ZV}}_{12}$$

where the coefficients are the eigenvectors of the quality indices, and F_1_, F_2_, and F_3_ are the scores of the principal components. The variance contribution rates βi (*i* = 1, 2, 3) of the initial eigenroots were used as the weighting coefficients of the first three principal components. Thereby, a quality evaluation model of the Luzhou‐flavor liquors, namely, the comprehensive score, was obtained in Equation (4):

(4) $$F=\frac{0\text{.51536}F1+0.36144F2+0.09691F3}{0.97371}$$

**TABLE 6:** Principal component load matrix and eigenvectors of the Luzhou‐flavor liquors

Index	First principal components		Second principal components		Third principal components	
	Loads	Eigenvectors	Loads	Eigenvectors	Loads	Eigenvectors
*V*_1_	−0.848	−0.341	0.372	0.179	0.310	0.287
*V*_2_	0.374	0.150	−0.033	−0.016	0.924	0.857
*V*_3_	−0.416	0.167	−0.467	−0.224	−0.720	−0.668
*V*_4_	0.858	0.345	−0.004	−0.002	0.468	0.434
*V*_5_	−0.106	−0.043	0.229	0.110	0.948	0.879
*V*_6_	−0.319	−0.128	0.932	0.448	0.085	0.079
*V*_7_	−0.169	−0.068	0.839	0.403	0.491	0.455
*V*_8_	−0.207	−0.083	0.973	0.467	−0.095	−0.088
*V*_9_	−0.352	−0.142	0.823	0.395	0.427	0.396
*V*_10_	0.942	0.379	−0.326	−0.157	−0.039	−0.036
*V*_11_	0.926	0.372	−0.156	−0.075	0.322	0.299
*V*_12_	0.919	0.370	−0.356	−0.171	0.154	0.143

The principal component matrix can also be used to measure the contributions of the principal components. Specifically, a larger absolute value of the load means that the contribution of the corresponding principal component is larger (Karytsas & Choropanitis, 2017). The first principal component has large loads in V_1_, V_4_, V_10_, V_11_, and V_12_, and mainly influences the liquor quality from the perspectives of ethyl caproate, ethyl butyrate, furfural, heptanoic acid, and octanoic acid (Table 6). The second principal component has large loads in V_6_, V_7_, V_8_, and V_9_, and mainly influences the liquor quality from the perspectives of 2‐methyl‐1‐propanol, butanol, isopentyl alcohol, and hexyl alcohol. The third principal component has large loads in V_2_, V_3_, and V_5_, and mainly influences the liquor quality from the perspectives of ethyl octanoate, ethyl lactate, and butyl caproate.

#### 3.2.3 Trace components by PCA

The comprehensive quality scores of Luzhou‐flavor liquors were determined using Equation (4) (Table 7). From the PCA‐based mathematical model, the scores of the five samples of Luzhou‐flavor liquors were ranked from high to low as M3, M1, M4, M2, and M5. Hence, the sensory evaluation of the five samples was M3 > M1 > M4 > M2 > M5.

**TABLE 7:** Comprehensive score of quality of Luzhou‐flavor liquor

Sample name	Scores of first principal components (*F*_1_)	Scores of second principal components (*F*_2_)	Scores of third principal components (*F*_3_)	Comprehensive score (*F*)	Rank
M1	−1.52	2.36	1.88	0.26	2
M2	−1.20	1.34	−1.92	−0.33	4
M3	3.00	−1.23	3.21	1.45	1
M4	−1.29	0.59	1.57	−0.30	3
M5	1.02	−3.06	−4.74	−1.07	5

### 3.3 Correlations of the Luzhou‐flavor liquor model

Correlations and significance between the model‐based comprehensive scores, F, and the sensory scores, were tested (Table 8). The correlation coefficients between the comprehensive scores and fuzzy mathematics sensory scores were up to 0.97, showing very high significance (*p* < .01), further validating the reliability of the evaluation model.

**TABLE 8:** Correlation between Luzhou‐flavor liquor model scores and sensory scores

Sample name	M1	M2	M3	M4	M5
Sensory scores	68.6	54.2	78.7	60.4	49.8
The model‐based comprehensive scores F	0.26	−0.33	1.45	−0.30	−1.07
Significance	*p* < .001				
Correlation coefficients	0.9717				

## 4 CONCLUSIONS

Fuzzy mathematics and PCA were used to comprehensively evaluate the Luzhou‐flavor liquors of different quality levels. Then, sensory evaluation data and trace components of the liquors were analyzed. Based on the modeling and data output, the liquor samples at close grades were ranked as M3 > M1 > M4 > M2 > M5. Thereby, a comprehensive liquor evaluation model has been established in this study. Compared to traditional liquor quality evaluation methods, this new method is more capable of performing comprehensive analysis and objective evaluation.

## CONFLICT OF INTEREST

The authors declare no conflict of interest.

## ETHICS STATEMENT

Our research did not contain any animal experiments or human subjects.

## Data Availability

Data available on request from the authors.
